# Biometric indicators of anterior segment parameters before and after laser peripheral iridotomy by swept-source optical coherent tomography

**DOI:** 10.1186/s12886-022-02448-1

**Published:** 2022-05-16

**Authors:** Bo Yu, Kang Wang, Xiaomin Zhang, Xiaoli Xing

**Affiliations:** Tianjin Key Laboratory of Retinal Functions and Diseases, Tianjin Branch of National Clinical Research Center for Ocular Disease, Eye Institute and School of Optometry, Tianjin Medical University Eye Hospital, Nankai District, No. 251, Fukang Road, Tianjin, 300384 China

**Keywords:** Primary angle closure, Laser peripheral iridotomy, Optical coherent tomography

## Abstract

**Background:**

Primary angle closure glaucoma (PACG) is the most common type of glaucoma in China. Laser peripheral iridotomy (LPI) is the primary choice to treat PAC: We aim to evaluate the changes of biometric parameters of anterior segment and to find possible biometric predictors of the effect of laser peripheral iridotomy (LPI) in primary angle closure (PAC) eyes using swept-source optical coherent tomography (OCT).

**Methods:**

LPI was performed in 52 PAC eyes of 28 participants. The change of intraocular pressure and anterior segment parameters, including angle opening distance (AOD500), AOD500 area, trabecular iris space area (TISA500), TISA500 volume, trabecular iris angle (TIA500), iridotrabecular contact (ITC) index, ITC area, anterior chamber volume (ACV), anterior chamber depth (ACD), lens vault (LV) and lens thickness (LT) before and 1 week after LPI were measured by Tomey CASIA2 anterior segment OCT. We also estimate and analyze potential associated factors possibly affecting the change of anterior chamber parameters.

**Results:**

No post-laser complications were found. The ACD, LV and LT did not change significantly 1 week after LPI. AOD500, AOD500 area, TISA500, TISA500 volume, TIA500, ACV increased significantly after LPI. There was significant decrease in ITC index and ITC area. LT was positively correlated to the change of ITC index (β = 0.239, *p = 0.045).

**Conclusions:**

The anterior segment architecture significantly changed after LPI in PAC spectrum eyes. Crystalline lens measurements remained unchanged before and after LPI. AS-OCT can be used to follow anterior chamber parameter changes in PAC spectrum eyes. LT may play a role in the therapeutic effect of LPI.

## Background

Glaucoma is the second most common cause of blindness worldwide. Primary angle closure glaucoma (PACG) accounts for 25% of all kinds of glaucoma globally. It is the most common type of glaucoma among Chinese descent [1]. When iridotrabecular contact (ITC) resulting in peripheral anterior synechiae (PAS) or raised IOP with no visual field defect or glaucomatous optic nerve damage are found, primary angle closure (PAC) can be diagnosed. It is an intermediate stage of the progression to PACG. Laser peripheral iridotomy (LPI) has been regarded as the primary choice to treat PAC by relieving pupillary block (PB) and widening the anterior chamber angle [2, 3]. However, LPI may not be satisfactory as long-term therapy in Asian eyes with acute.

PAC [4]. The Academy of American Ophthalmology even demonstrated the superiority of cataract surgery over LPI in acute PAC [5]. Considering health economy, individual income and opportunity cost, widespread prophylactic LPI for PAC suspects is not recommended [6].

In clinic, gonioscopy is the gold standard to diagnose PAC. However, gonioscopy doesn’t possess the ability to quantitatively measure anterior chamber parameters. Ultrasound biomicroscopy (UBM) and anterior segment optical coherence tomography (AS-OCT) can be used to characterize LPI-induced anatomical changes quantitatively [7]. Compared with AS-OCT, UBM is a contact and time-consuming imaging modality with supine position required during examination. So AS-OCT is widely used to obtain high-resolution images of anterior chamber [8]. It has undergone several generations of innovation from time-domain OCT (TD-OCT), spectral-domain OCT (SD-OCT) to the swept-source OCT (SS-OCT). The CASIA2 SS-AS-OCT, with a high scan speed, provides a higher sensitivity for depth and higher resolution pictures. It has made it possible to obtain parameter data from cornea to posterior lens in one image just in a few seconds.

To find more conventional measurement index to guide clinical LPI treatment, lens parameters were chosen instead of iris parameters. In the current study, we compared the anterior chamber, anterior chamber angle and lens parameters before and after LPI, aiming to demonstrate the effectiveness of LPI in PAC eyes and to examine baseline anterior chamber, anterior chamber angle and lens parameters associated with the effectiveness of LPI in Chinese population.

## Methods

### Study design and patient enrollment

This is a prospective, hospital-based, observational study that was approved by the Ethics Committee of Tianjin Medical University Eye Hospital [202KY(L)-52]. The study enrolled 28 patients (52 eyes), who underwent LPI for PAC state at the glaucoma clinic of Tianjin Medical University Eye Hospital in April 2020. The informed consent was signed before entering the study.

All enrolled eyes fulfilled the diagnosis criteria of PAC 1. Narrow angles were found by gonioscopy (at least 180° of trabecular meshwork was invisible, Spaeth grade A or B); 2. IOP > 21 or normal IOP with IOP lowering drugs used 3. PAS was found; 4. No glaucomatous damage of the optic nerve was found (visual field was normal and vertical cup-to-disc ratio was no more than 0.6). The exclusion criteria were as follows: 1. subjects unable to tolerate LPI treatment, for example, those with severe systemic diseases; 2. subjects unable to cooperate well with the conductor to receive all the examinations; 3. subjects not suitable for gonioscopy, for example, those with infectious conjunctivitis; 4. eyes with apparent ocular diseases except for cataract; 5. eyes with a history of laser procedure or intraocular surgery; 6. eyes diagnosed as secondary angle closure; 7. eyes with acute PAC or other conditions which needed surgery intervention.

All participants underwent baseline ophthalmic examination including slit-lamp biomicroscopy, non-mydriatic fundus examination, gonioscopy, Goldmann applanation tonometer, visual field test. Slit-lamp biomicroscopy was done by a trained ophthalmologist to exclude subjects with other ocular diseases or abnormal cup-to-disc ratio. Static and dynamic gonioscopy were performed by an experienced gonioscopist to assess the condition of anterior chamber angle, to diagnose PAC and to identify the presence of PAS (illumination about 90 lx). The angle widths were graded using the Spaeth gonioscopic grading scale: A. open to Schwalbe’s line; B. open to anterior trabecular meshwork; C. open to posterior trabecular meshwork; D. open to scleral spur; E. open to ciliary body band in each of 4 quadrants [9]. Visual field tests were done with the 24–2 pattern on the Humphrey Field Analyzer (Carl Zeiss Meditec, Dublin, CA). To avoid learning effect, the second reliable visual field result was accepted as the final one. Visual field results were considered abnormal if one of the following criteria was met having a glaucoma hemifield test “outside normal limits”, or pattern standard deviation (PSD) at a *P* < 5% level, or a cluster of three non-edge locations worse than a P level of 5% with at least one worse than a P level of 1% on the pattern deviation plot (PDP) [10]. The IOP lowering drugs used by the subjects were not strictly controlled.

LPI was performed after all baseline examination being finished. Before LPI, 1% pilocarpine nitrate eye drops were given four times in half an hour. IOP was measured again 20 min after LPI. Prednisolone acetate and pranoprofen eye drops were administered 4 times a day for a week. To ensure the consistency of each subject before and after LPI treatment, IOP lowering drugs administration remained unchanged during the whole experimental process. AS-OCT were performed before and 1 week after LPI for each subject. IOP was measured again 1 week after LPI.

### Image acquisition and parameter measurement

AS-OCT was performed by an experienced ophthalmic imaging technician. All subjects underwent AS-OCT (Tomey CASIA2) under ambient room lighting conditions (illumination around 90 lx). The CASIA2 uses a 1310 nm swept-source laser wavelength at a frequency of 0.3 s. Consecutive scans were performed under “anterior chamber angle” mode. Sixteen meridian images were acquired. All patients had an AS-OCT scan performed by the same technician 1 week after LPI treatment.

The obtained images were analyzed by the software provided by the manufacturer. This build-in software can automatically analyze the anterior segment structures and provide measurement results after scleral spurs (SSs) were marked. The SS was defined as the inward protrusion of the sclera where a change in curvature of the corneoscleral junction was observed. A trained technician who was masked to the gonioscopic grading marked SSs according to the rule. A total of 8 equally-spaced angle images were chosen to estimate the average angle parameters [11]. The analyzed parameters included average angle opening distance (AOD500, defined as AOD at 500 μm from the marked SSs), AOD500 area (defined as the area formed by 360° AOD500), average trabecular iris space area (TISA500, defined as the area formed by the following four points: trabecular meshwork at 500 μm from the marked SSs, angle recess, intersection of trabecular meshwork at 500 μm from the marked SSs perpendicular line and iris anterior surface, intersection of angle recess perpendicular line and iris anterior surface), TISA500 volume, (defined as the volume formed by 360° TISA500), trabecular iris angle (TIA500, defined as the angle formed by trabecular meshwork at 500 μm from the marked SSs, angle recess and intersection of trabecular meshwork at 500 μm from the marked SSs perpendicular line and iris anterior surface), iridotrabecular contact index (ITCI, defined as the percentage of iridotrabecular contact areas to all measured areas), ITC area, anterior chamber volume (ACV), anterior chamber depth (ACD), lens vault (LV, defined as the distance between the intersection of perpendicular bisector of SSs connection and lens and the midpoint of SSs connection) and lens thickness (LT).

### Statistical analysis

Statistical analysis was performed by SPSS (Version 22). Continuous data were presented as mean ± SD. Parameter variable before and after LPI analysis was performed using Wilcoxon signed-rank test when data distribution did not accord with normal distribution, or paired *t* test when data distribution followed normal distribution. *P* < 0.01 was considered statistically significant. All data was standardized to reduce dimensional differences before entering regression analysis. Variables were first analyzed on the univariate model. *P* < 0.05 was considered statistically significant. Then multiple logistic regression model was established to analyze the variables that were statistically significant.

## Results

Fifty-two eyes of 28 PAC patients that qualified for the initial inclusion criteria were included in our study. Characteristics of the enrolled subjects at baseline were shown as follows. Among 28 patients 9 were male (16 eyes). The mean age of the subjects was 63.65 ± 6.04 years old with the range of 54 years old to 74 years old. The mean IOP pre-LPI was 17.35 ± 3.61 mmHg. None of the patients had experienced post-LPI complications such as persistent uveitis, persistent and serious IOP elevation or persistent pain. Among 52 eyes, 22 eyes were given pilocarpine; 17 eyes were given carteolol hydrochloride; 9 eyes were given brinzolamide; 9 eyes were given brimonidine before enrollment. IOP lowering drugs used by the patients remained unchanged before and after LPI.

There was a statistically significant increase in AOD500, AOD500 area, TISA500, TISA500 volume, TIA500 and ACV before and 1 week after LPI. ITCI and ITC area decreased significantly post-LPI. There was no statistical significance in ACD, LV and LT before and 1 week after LPI. IOP increased significantly 15 min after LPI, while had a small but significant decrease 1 week after LPI. The results were shown in Table 1.

**Table 1:** Anterior chamber and angle parameters measured by AS-OCT before and 1 week after LPI

	Pre-LPI	One week post LPI	*p-*value
AOD500 (mm)	0.125 ± 0.080	0.257 ± 0.106	< 0.01^*^
AOD500 area (mm^2^)	3.978 ± 2.515	7.942 ± 3.336	< 0.01^*^
TISA500 (mm^2^)	0.045 ± 0.031	0.110 ± 0.129	< 0.01^*^_#_
TISA500 volume (mm^3^)	1.594 ± 1.162	3.163 ± 1.344	< 0.01^*^_#_
TIA500 (°)	12.998 ± 7.980	24.831 ± 9.476	< 0.01^*^
ITCI (%)	41.440 ± 29.468	16.206 ± 17.266	< 0.01^*^
ITC area (mm^2^)	6.399 ± 6.119	1.739 ± 2.977	< 0.01^*^_#_
ACD (mm)	1.818 ± 0.262	1.859 ± 0.239	0.066
ACV (mm^3^)	68.946 ± 16.112	85.055 ± 14.711	< 0.01^*^
LT (mm)	5.041 ± 0.288	5.007 ± 0.290	0.045
LV (mm)	0.898 ± 0.333	0.884 ± 0.253	0.431

AOD500 Angle opening distance at 500 μm anterior to the scleral spur, TISA500 Trabecular iris space area at 500 μm anterior to the scleral spur, TIA500 Trabecular iris angle at 500 μm anterior to the scleral spur, *ACV* Anterior chamber volume, *ACD* Anterior chamber depth), *LV* Lens vault and *LT* Lens thickness

^*^Statistically significant

^#^ The data distribution followed normal distribution and paired *t* test was used. The other data were analyzed by Wilcoxon signed-rank test

In the univariate regression analysis, the primary IOP, AOD500, TISA500, TIA500, ACD and ACV were negatively correlated with the change of ITCI before and after LPI treatment in PAC eyes. LT was positively correlated with the change of ITCI (*p* < 0.05^*^). In the multiple regression analysis. LT weas proved to be positively correlated with the change of ITCI (*p* = 0.045^*^). The results were shown in Table 2.

**Table 2:** Univariate and multiple logistic regression analysis of the correlation between pre-LPI parameters and the changes of ITCI

	Univariate regression analysis		Multiple regression analysis	
	Standardized β (95% CI)	*p*-value	Standardized β (95% CI)	*p*-value
IOP (mmHg)^a^	-0.520 (-0.772, -0.268)	< 0.001	-0.163 (-0.383, 0.058)	0.148
AOD500 (mm) ^a^	-0.66 (-0.908, -0.412)	< 0.001	-0.904 (-2.052, 0.244)	0.123
TISA500 (mm^2^) ^a^	-0.626 (-0.872, -0.279)	< 0.001	0.992 (-0.316, 2.300)	0.137
TIA500 (°)^a^	-0.656 (-0.913, -0.398)	< 0.001	-0.706 (-2.176, 0.765)	0.347
ACD (mm) ^a^	-0.292 (-0.503, -0.081)	0.007	0.130 (-0.288, 0.371)	0.542
ACV (mm^3^) ^a^	-0.318(-0.569, -0.067)	0.013	0.149 (-0.395, 0.693)	0.591
LT (mm) ^a^	0.411 (0.181, 0.642)	< 0.001	0.239 (0.005, 0.472	0.045^*^
LV (mm) ^a^	0.269 (-0.027, 0.565)	0.075	-	-

*IOP* Intraocular pressure, AOD500 Angle opening distance at 500 μm anterior to the scleral spur, TISA500 Trabecular iris space area at 500 μm anterior to the scleral spur, TIA500 Trabecular iris angle at 500 μm anterior to the scleral spur, *ACV* Anterior chamber volume, *ACD* Anterior chamber depth, *LV* Lens vault and *LT* Lens thickness

^*^ Statistically significant from multiple regression analysis, *CI* Confidence interval

^a^ Data has been standardized

## Discussion

The current study utilized Tomey CASIA2 AS-OCT to assess changes of anterior segment parameters after LPI in PAC eyes of Chinese descent. Compared with former types of AS-OCT, CASIA2, with high scanning speed and its built-in program, made the measurement of the average values of 360° anterior chamber parameters in a few seconds possible. Similar with former studies, our results revealed that most anterior chamber parameters, such as AOD, TISA, TIA and ACV changed after LPI, demonstrating the therapeutic effect of LPI [12, 13]. We also found that ITCI and ITC area also changed. They are 360° indicators calculated by the built-in analysis software according to the ITC condition of the 16 meridian images, which reflected angle closure visually. ITCI have been validated in the evaluation of circumferential angle closure in previous studies and been proved to be feasible [14, 15].

In order to explore the effects of LPI, we analyzed the correlation between angle and lens biological parameters and ICTI. We found that the change of ITCI before and after LPI is positively correlated with LT, which has not been reported before. It has been reported that LT can be regarded as predictable factors in the occurrence of PACG [16, 17]. Moreover, the ITCI, as an indicator of the stages of PAC, was reported to be positively correlated with LT [18]. When the lens has greater thickness, a greater curve of its anterior surface with aging, the possibility of PB increases. PB is considered as the key element in the pathogenesis of PAC [19, 20]. LPI is often performed to alleviate PB. So we think PAC eyes with thicker lens are more suitable for LPI treatment.

Some studies focused on the same point as we do in order to find possible predictors of the angle widening degree after LPI. Iris parameters were related to the change of the angle closure indicators. Some studies reported that greater iris curvature was predictive of greater angle widening [13, 21, 22]. Different from the above research, iris parameters were not included in the current study. Iris and lens parameters are closely inter-correlated. Iris parameters might play more important roles in the prediction of greater angle widening. However, iris parameters can only be measured by AS-OCT or UBM. Lens parameters are more convenient and economical to acquire. We excluded the iris parameters in the analysis to enlarge the influence of lens parameters.

Tomey CASIA2 AS-OCT offers reliable quantitative measurements of anterior segment structures [23]. Formerly used standard anterior segment OCT measures angle parameters in a single section. SS-OCT allows 3-dimensional measurements of angle parameters, such as the circumferential ITCI, ITC area and AOD area. Previously, we often used UBM to quantitatively assess angle parameters. However, AS-OCT possesses some advantages over UBM. Higher degree of resolution, higher scan speed and the noncontact feature made the examination more comfortable, faster and safer for the patients. It is easier to learn and operate for the ophthalmic imaging technician. With a high scan speed of 50,000 A-scans per second, it takes only 0.016 s to capture a single cross-sectional image, making the 360° measurement of anterior chamber angle parameters done in just 2.4 s [24]. Considering the limitation of previously used instruments, most former studies only assessed the change of nasal and/or temporal anterior chamber angle parameters [12, 25]. We measured average parameters around 360° anterior segment using the built-in program in the current study, which provided more reliable data.

LPI is widely used as a preventative treatment to PAC suspects and used to treat PAC and PACG patients without doubts before [26]. However, EAGLE (Effectiveness of Early Lens Extraction for the Treatment of Primary Angle-Closure Glaucoma) study reported that early lens extraction is cost-effectively preferred over LPI in the management of PACG [27]. Another study reported that clear lens extraction resulted in a more significant effect in widening of the anterior chamber angle in PAC eyes without cataract than LPI [28]. Although these studies pointed out the benefits of clear lens extraction in PAC and PACG eyes, debates still exist. Considering the relatively short follow-up time, long-term benefits and safety of clear lens extraction should be further evaluated. It takes a long time and a lot of money to train qualified and experienced surgeons to do the surgery. They should master the procedure expertly and possess the ability to deal with all complications timely and properly [29]. So currently, in developing countries, the role of LPI in the management of PAC eyes is still irreplaceable. Jiang et al. found that the drainage angle widened at 2 weeks after LPI in treated eyes [30]. He et al. also found LPI resulted in a significant increase in angle width in individuals with narrow angles [31]. Lin et al. reported that angle width changed at 2 weeks after LPI in narrow angle eyes without peripheral anterior synechia (PAS) [2]. So LPI was shown to be effective in treating PAC spectrum diseases in Chinese population-based studies.

Due to the wide use of LPI, some research focused on finding the baseline factors possibly influencing the outcome of it. A New Zealand research found that pre-laser greater angle closure and less anterior iris bowing predicted the failure of LPI [32]. Another study reported that in South Indian population with PACS or PAC/ PACG, greater postoperative angle widening was associated with greater baseline PB, such as shorter AOD750, shorter axial length and greater lens vault [13]. PAS also influence the successful rate of LPI [2]. Baskaran et al. found that greater iris volume and higher IOP are risk factors for residual angle closure after LPI [33]. In the current study, we use the change of ITCI before and one week after LPI to evaluate the short-term effect of it. Interestingly, we find LT correlates with the change of ITCI.

This study has a few limitations. First, the sample size is small. More subjects need to be enrolled to verify the current results. Second, the follow-up time is short. Our study only evaluates the shot-term effect of LPI. Long-term side effects and the effectiveness should be studied further. Third, factors such as the time of IOP measurement are not strictly controlled, which might increase the deviation of the parameters. Fourth, IOP lowering drug administration is not consistent among different subjects. The use of pilocarpine might affect some of the parameters. Last, all examinations are performed in the environment with light on, not in the dark.

## Conclusions

Our study describes the average parameters around 360° anterior segment changes before and short-term after LPI. Anterior chamber angle widens and ITC index decreases 1 week after LPI. When evaluating baseline parameters potentially affecting ITCI change, we find that LT is positively correlated with it. Our results describe the anatomic changes reflected by AS-OCT before and after LPI. PAC patients with thicker lens might be more suitable for LPI treatment. Tomey CASIA2 AS-OCT produces convenient and reliable measurement for anterior segment parameters in clinic.

## Data Availability

The datasets used and analyzed during the current study are available from the corresponding author on reasonable request.
